# *tert*-Butyl *N*-[3-hy­droxy-1-phenyl-4-(pyrimidin-2-ylsulfan­yl)butan-2-yl]carbamate monohydrate

**DOI:** 10.1107/S1600536811031850

**Published:** 2011-08-11

**Authors:** Claudia R. B. Gomes, Thatyana R. A. Vasconcelos, Walcimar T. Vellasco, James L. Wardell, Solange M. S. V. Wardell, Edward R. T. Tiekink

**Affiliations:** a Instituto de Tecnologia em Fármacos - Farmanguinhos, FioCruz –, Fundação Oswaldo Cruz, R. Sizenando Nabuco, 100, Manguinhos, 21041-250 Rio de Janeiro, RJ, Brazil; b Universidade Federal Fluminense, Instituto de Química, Departamento de Química Orgânica, Outeiro de São João Batista, s/no, Centro, Niterói, 24020-141 Rio de Janeiro, Brazil; c Centro de Desenvolvimento Tecnológico em Saúde (CDTS), Fundação Oswaldo Cruz (FIOCRUZ), Casa Amarela, Campus de Manguinhos, Av. Brasil 4365, 21040-900 Rio de Janeiro, RJ, Brazil; d CHEMSOL, 1 Harcourt Road, Aberdeen AB15 5NY, Scotland; e Department of Chemistry, University of Malaya, 50603 Kuala Lumpur, Malaysia

## Abstract

In the title hydrate, C_19_H_25_N_3_O_3_S·H_2_O, the configuration at each chiral centre in the organic mol­ecule is *S*, with the hy­droxy and carbamate substituents being *anti* [O—C—C—N torsion angle = −179.3 (3)°]. The thio­pyrimidyl and carbamate residues lie to one side of the pseudo-mirror plane defined by the C_5_S backbone of the mol­ecule; this plane approximately bis­ects the benzene ring at the 1- and 4-C atoms. The dihedral angle formed between the terminal rings is 5.06 (18)°. In the crystal, supra­molecular tubes aligned along the *b* axis are found: these are sustained by a combination of O—H⋯O, O—H⋯N and N—H⋯O hydrogen bonds.

## Related literature

For background to the use of hy­droxy­ethyl­amine derivatives in medicinal chemistry, see: Brik & Wong (2003 ▶); Ghosh *et al.* (2001 ▶); Marcin *et al.* (2011 ▶); Trudel *et al.* (2008 ▶); Cunico *et al.* (2009*a* ▶,*b* ▶,*c* ▶, 2011 ▶).
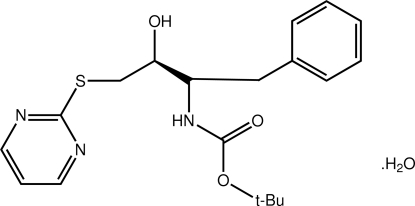


## Experimental

### 

#### Crystal data

C_19_H_25_N_3_O_3_S·H_2_O
*M* *_r_* = 393.50
Monoclinic, 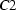

*a* = 19.4238 (7) Å
*b* = 5.1275 (2) Å
*c* = 22.4815 (8) Å
β = 114.319 (2)°
*V* = 2040.38 (13) Å^3^
*Z* = 4
Mo *K*α radiation
μ = 0.19 mm^−1^
*T* = 120 K
0.30 × 0.02 × 0.02 mm

#### Data collection

Bruker–Nonius Roper CCD camera on κ-goniostat diffractometer
Absorption correction: multi-scan (*SADABS*; Sheldrick, 2007 ▶) *T*_min_ = 0.801, *T*_max_ = 1.000
11781 measured reflections
4032 independent reflections
3409 reflections with *I* > 2σ(*I*)
*R*_int_ = 0.048

#### Refinement

*R*[*F* ^2^ > 2σ(*F* ^2^)] = 0.055
*wR*(*F* ^2^) = 0.112
*S* = 1.04
4032 reflections
259 parameters
6 restraints
H atoms treated by a mixture of independent and constrained refinement
Δρ_max_ = 0.27 e Å^−3^
Δρ_min_ = −0.26 e Å^−3^
Absolute structure: Flack (1983 ▶), 1442 Friedel pairs
Flack parameter: 0.11 (11)

### 

Data collection: *COLLECT* (Hooft, 1998 ▶); cell refinement: *DENZO* (Otwinowski & Minor, 1997 ▶) and *COLLECT*; data reduction: *DENZO* and *COLLECT*; program(s) used to solve structure: *SHELXS97* (Sheldrick, 2008 ▶); program(s) used to refine structure: *SHELXL97* (Sheldrick, 2008 ▶); molecular graphics: *ORTEP-3* (Farrugia, 1997 ▶) and *DIAMOND* (Brandenburg, 2006 ▶); software used to prepare material for publication: *publCIF* (Westrip, 2010 ▶).

## Supplementary Material

Crystal structure: contains datablock(s) global, I. DOI: 10.1107/S1600536811031850/hb6348sup1.cif

Structure factors: contains datablock(s) I. DOI: 10.1107/S1600536811031850/hb6348Isup2.hkl

Supplementary material file. DOI: 10.1107/S1600536811031850/hb6348Isup3.cml

Additional supplementary materials:  crystallographic information; 3D view; checkCIF report

## Figures and Tables

**Table 1:** Hydrogen-bond geometry (Å, °)

*D*—H⋯*A*	*D*—H	H⋯*A*	*D*⋯*A*	*D*—H⋯*A*
O1—H1o⋯O1w^i^	0.83 (2)	2.00 (2)	2.813 (4)	168 (4)
O1w—H1w⋯N1^ii^	0.85 (2)	2.12 (2)	2.958 (4)	174 (4)
O1w—H2w⋯O1	0.84 (3)	2.06 (3)	2.893 (4)	170 (4)
N3—H3n⋯O2^i^	0.86 (2)	2.13 (2)	2.910 (3)	152 (3)

Symmetry codes: (i) 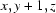; (ii) 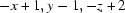.

**Fig. 1.**
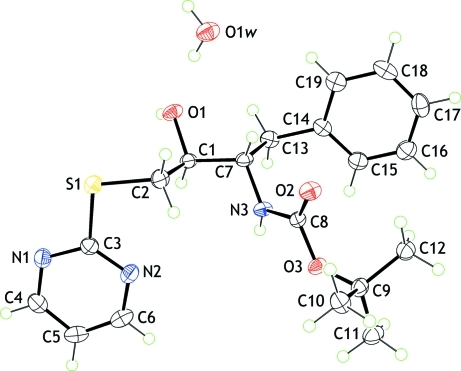
The molecular structure of (I) showing displacement ellipsoids at the 50% probability level.

**Fig. 2.**
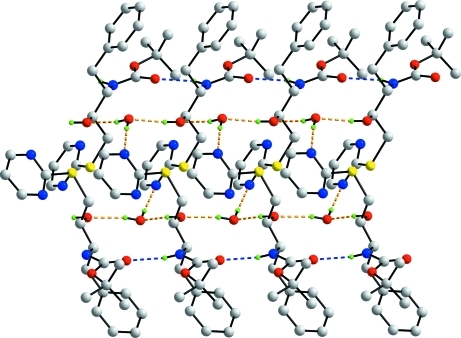
A side-on view of the supramolecular tube in (I) sustained by O—H···O, O—H···N (orange dashed lines) and N—H···O (blue dashed lines) hydrogen bonds.

**Fig. 3.**
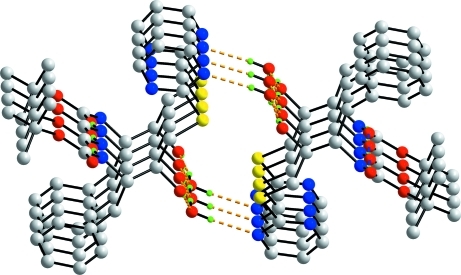
End-on view of the supramolecular tube in (I) sustained by O—H···O, O—H···N (orange dashed lines) and N—H···O (blue dashed lines) hydrogen bonds.

**Fig. 4.**
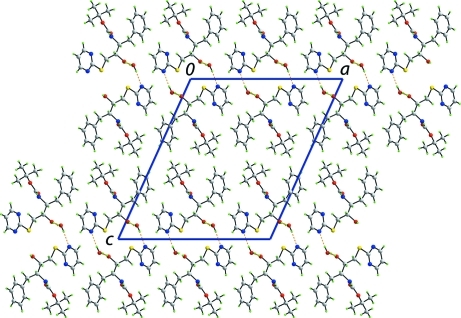
A view in projection down the b axis of the packing of supramolecular tubes in (I). The O—H···O, O—H···N (orange) and N—H···O (blue) hydrogen bonds are shown as dashed lines.

## supplementary crystallographic information

### Comment

Compounds having a hydroxyethylamine core play important roles in the medicinal chemistry field. They inhibit aspartyl protease enzymes and are widely used as anti-HIV agents (Brik & Wong, 2003; Ghosh *et al.*, 2001), as inhibitors of BACE-1 to combat Alzheimer's disease (Marcin, *et al.*, 2011) and have also been considered in the treatment of leishmania/HIV-1 co-infections (Trudel *et al.*, 2008). Cunico and co-workers have reported on the *in vitro* activity of hydroxyethylamine derivatives as anti-malarial agents (Cunico *et al.*, 2009*a*, 2009*b*, 2009*c*, 2011) and in this article we report the structure of the title molecule, isolated from ethanol solution as a monohydrate, (I).

The structure analysis of (I) confirms the stereochemistry at each of the C1 and C7 atoms to be *S*, Fig. 1, as anticipated from the synthesis. The O1 and N3 substituents on C1 and C7, respectively, have an *anti* disposition [the O1—C1—C7—N3 torsion angle = -179.3 (3) °]. With reference to the C_5_S backbone of the molecule, *i.e*. comprising the C1/C2/C7/C13/C14/S1 atoms, the benzene ring occupies a position that is approximately bisected by the pseudo mirror plane through these atoms [the C7—C13—C14···C17 torsion angle = -17.1 (5) °] whereas the thiopyrimidyl group lies to one side of the plane [the C3—S1—C2—C1 torsion angle = 89.9 (2) °]. The terminal rings of the C_5_S backbone are almost parallel forming a dihedral angle of 5.06 (18) °. The carbamate residue lies to the same side of the C_5_S plane as does the thiopyrimidyl group with the *t*-BuO atoms being directed away from the rest of the molecule.

In the crystal, the water molecules serve to link translationally related hydroxy groups by forming both donor and acceptor interactions, and at the same time the water molecule forms a donor interaction to one of the pyrimidyl-N atoms, Table 1. The resultant supramolecular assembly, a tube, is further stabilized by amine-*H*···*O*(carbonyl) interactions. Side-on and end-on views of the supramolecular tube are shown in Figs 2 and 3, respectively. The tubes are aligned along the *b* direction as seen in Fig. 4.

### Experimental

A solution of (2*S*,3*S*)-boc-phenylalanine epoxide (1.6 mmol) (Cunico *et al.*, 2009*a*), mercaptopyrimidine (1.5 mmol) and triethylamine (1.6 mmol) in methanol (10 ml) was stirred at room temperature for 2 h, rotary evaporated and HCl (5%) added to the residue. The mixture was extracted with CH_2_Cl_2_ and the combined organic extracts were washed with brine, dried over anhydrous Na_2_SO_4_ and evaporated, giving the title molecule in 95% yield. The crude product was purified by crystallization in methanol/water (7:3) to yield colourless needles of (I); *M*.pt.: 371–373 K.. EI—MS (m/*z*) (%): 398.2 (*M*++Na, 52%). ^1^H NMR [400.00 MHz, DMSO-d6] δ: 8.59 (d, 2H, J = 4.8 Hz, H3' and H5'); 7.18 (t, 1H, J = 4.7 Hz, H4'); 7.19–7.13 (m, 5H, Ph); 6.70 (d, 1H, J = 8.7 Hz, NH); 5.33 (d, 1H, J = 6.0 Hz, OH); 3.66–3.58 (m, 2H, H3 and H2); 3.52 (dd, 1H, ^1^J = 13.6 Hz, ^2^J = 3.2 Hz, H1b); 3.08 (dd, 1H, ^1^J = 13.6, ^2^J = 8.0 Hz, H1a); 3.03 (dd, 1H, ^1^J = 13.5, ^2^J = 2.6 Hz, H4b); 2.56 (dd, 1H, ^1^J = 13.7, 2 J = 10.0 Hz, H4a); 1.26 (s, 9H, Boc) p.p.m.. ^13^C NMR [100.0 MHz, DMSO-d6] δ: 171.4; 157.6; 155.3; 139.5; 129.1; 127.9; 125.7; 117.0; 77.5; 72.1; 56.3; 35.8; 35.0; 28.2 p.p.m.. IR (cm^-1^; KBr): ν_max_: 3358 (OH); 3030 (NH); 1686 (C═O); 640 (C—S). The crystals used in the structure determination were grown from moist EtOH solution explaining the presence of water in the title structure, (I).

### Refinement

The C-bound H atoms were geometrically placed (C–H = 0.95–1.00 Å) and refined as riding with *U*_iso_(H) = 1.2–1.5*U*_eq_(C). The O– and N-bound H atoms were located from a difference map and refined with the distance restraints O–H = 0.84 ± 0.01 and N–H = 0.86±0.01 Å, and with *U*_iso_(H) = *zU*_eq_(carrier atom); *z* = 1.5 for O and *z* = 1.2 for N.

### Crystal data

**Table:**

C_19_H_25_N_3_O_3_S·H_2_O	*F*(000) = 840
*M**_r_* = 393.50	*D*_x_ = 1.281 Mg m^−^^3^
Monoclinic, *C*2	Mo *K*α radiation, λ = 0.71073 Å
Hall symbol: C 2y	Cell parameters from 31450 reflections
*a* = 19.4238 (7) Å	θ = 2.9–27.5°
*b* = 5.1275 (2) Å	µ = 0.19 mm^−^^1^
*c* = 22.4815 (8) Å	*T* = 120 K
β = 114.319 (2)°	Needle, colourless
*V* = 2040.38 (13) Å^3^	0.30 × 0.02 × 0.02 mm
*Z* = 4	

### Data collection

**Table:**

Bruker–Nonius Roper CCD camera on κ-goniostat diffractometer	4032 independent reflections
Radiation source: Bruker-Nonius FR591 rotating anode	3409 reflections with *I* > 2σ(*I*)
graphite	*R*_int_ = 0.048
Detector resolution: 9.091 pixels mm^-1^	θ_max_ = 27.5°, θ_min_ = 2.9°
φ and ω scans	*h* = −24→24
Absorption correction: multi-scan (*SADABS*; Sheldrick, 2007)	*k* = −6→5
*T*_min_ = 0.801, *T*_max_ = 1.000	*l* = −29→28
11781 measured reflections	

### Refinement

**Table:**

Refinement on *F*^2^	Secondary atom site location: difference Fourier map
Least-squares matrix: full	Hydrogen site location: inferred from neighbouring sites
*R*[*F*^2^ > 2σ(*F*^2^)] = 0.055	H atoms treated by a mixture of independent and constrained refinement
*wR*(*F*^2^) = 0.112	*w* = 1/[σ^2^(*F*_o_^2^) + (0.0169*P*)^2^ + 4.9933*P*] where *P* = (*F*_o_^2^ + 2*F*_c_^2^)/3
*S* = 1.04	(Δ/σ)_max_ = 0.001
4032 reflections	Δρ_max_ = 0.27 e Å^−^^3^
259 parameters	Δρ_min_ = −0.26 e Å^−^^3^
6 restraints	Absolute structure: Flack (1983), 1442 Friedel pairs
Primary atom site location: structure-invariant direct methods	Flack parameter: 0.11 (11)

### Special details

**Table:**

Geometry. All s.u.'s (except the s.u. in the dihedral angle between two l.s. planes) are estimated using the full covariance matrix. The cell s.u.'s are taken into account individually in the estimation of s.u.'s in distances, angles and torsion angles; correlations between s.u.'s in cell parameters are only used when they are defined by crystal symmetry. An approximate (isotropic) treatment of cell s.u.'s is used for estimating s.u.'s involving l.s. planes.
Refinement. Refinement of *F*^2^ against ALL reflections. The weighted *R*-factor *wR* and goodness of fit *S* are based on *F*^2^, conventional *R*-factors *R* are based on *F*, with *F* set to zero for negative *F*^2^. The threshold expression of *F*^2^ > 2σ(*F*^2^) is used only for calculating *R*-factors(gt) *etc*. and is not relevant to the choice of reflections for refinement. *R*-factors based on *F*^2^ are statistically about twice as large as those based on *F*, and *R*- factors based on ALL data will be even larger.

### Fractional atomic coordinates and isotropic or equivalent isotropic displacement parameters (Å^2^)

**Table:**

	*x*	*y*	*z*	*U*_iso_*/*U*_eq_	
S1	0.38657 (5)	0.93234 (19)	0.94519 (4)	0.0274 (2)	
O1	0.51141 (12)	0.8689 (5)	0.89189 (11)	0.0260 (6)	
H1O	0.526 (2)	1.023 (3)	0.898 (2)	0.039*	
O2	0.33859 (13)	0.2943 (4)	0.71553 (11)	0.0262 (6)	
O3	0.25342 (12)	0.5902 (4)	0.64894 (11)	0.0230 (5)	
N1	0.29924 (15)	1.2998 (6)	0.95196 (13)	0.0263 (7)	
N2	0.27041 (16)	1.1435 (6)	0.84405 (13)	0.0274 (7)	
N3	0.35124 (14)	0.7325 (5)	0.73525 (13)	0.0195 (6)	
H3N	0.3325 (17)	0.881 (3)	0.7189 (14)	0.023*	
C1	0.43449 (16)	0.8764 (7)	0.84546 (14)	0.0212 (7)	
H1	0.4184	1.0614	0.8337	0.025*	
C2	0.38603 (18)	0.7501 (7)	0.87609 (15)	0.0247 (7)	
H2A	0.4049	0.5714	0.8905	0.030*	
H2B	0.3334	0.7358	0.8428	0.030*	
C3	0.31002 (17)	1.1467 (7)	0.90796 (16)	0.0228 (7)	
C4	0.24166 (18)	1.4693 (7)	0.92708 (16)	0.0300 (8)	
H4	0.2315	1.5825	0.9560	0.036*	
C5	0.19690 (19)	1.4849 (8)	0.86141 (17)	0.0337 (9)	
H5	0.1561	1.6048	0.8445	0.040*	
C6	0.21382 (19)	1.3195 (8)	0.82139 (17)	0.0324 (9)	
H6	0.1844	1.3292	0.7757	0.039*	
C7	0.42887 (16)	0.7273 (7)	0.78451 (14)	0.0203 (7)	
H7	0.4438	0.5418	0.7969	0.024*	
C8	0.31684 (17)	0.5180 (6)	0.70118 (15)	0.0187 (7)	
C9	0.20689 (17)	0.3927 (7)	0.60200 (15)	0.0232 (7)	
C10	0.16727 (19)	0.2235 (7)	0.63384 (17)	0.0282 (8)	
H10A	0.1391	0.3348	0.6514	0.042*	
H10B	0.2049	0.1222	0.6693	0.042*	
H10C	0.1323	0.1045	0.6013	0.042*	
C11	0.15008 (19)	0.5600 (7)	0.54834 (17)	0.0292 (8)	
H11A	0.1767	0.6684	0.5286	0.044*	
H11B	0.1231	0.6722	0.5669	0.044*	
H11C	0.1138	0.4472	0.5149	0.044*	
C12	0.25420 (18)	0.2360 (7)	0.57476 (16)	0.0260 (8)	
H12A	0.2829	0.1024	0.6064	0.039*	
H12B	0.2892	0.3529	0.5665	0.039*	
H12C	0.2208	0.1520	0.5338	0.039*	
C13	0.48159 (17)	0.8439 (7)	0.75607 (15)	0.0241 (7)	
H13A	0.4615	1.0151	0.7361	0.029*	
H13B	0.5320	0.8734	0.7919	0.029*	
C14	0.48983 (18)	0.6690 (7)	0.70518 (16)	0.0227 (7)	
C15	0.44298 (19)	0.6935 (7)	0.63926 (16)	0.0274 (8)	
H15	0.4064	0.8291	0.6250	0.033*	
C16	0.44908 (19)	0.5222 (7)	0.59414 (17)	0.0298 (8)	
H16	0.4163	0.5397	0.5492	0.036*	
C17	0.50219 (19)	0.3270 (8)	0.61384 (17)	0.0316 (8)	
H17	0.5059	0.2091	0.5827	0.038*	
C18	0.55023 (19)	0.3022 (7)	0.67897 (18)	0.0321 (8)	
H18	0.5876	0.1693	0.6926	0.038*	
C19	0.54376 (18)	0.4719 (7)	0.72444 (17)	0.0279 (8)	
H19	0.5766	0.4532	0.7693	0.033*	
O1W	0.58202 (13)	0.3603 (5)	0.91425 (12)	0.0322 (6)	
H1W	0.6181 (15)	0.351 (8)	0.9517 (9)	0.048*	
H2W	0.561 (2)	0.506 (4)	0.912 (2)	0.048*	

### Atomic displacement parameters (Å^2^)

**Table:**

	*U*^11^	*U*^22^	*U*^33^	*U*^12^	*U*^13^	*U*^23^
S1	0.0276 (4)	0.0302 (5)	0.0216 (4)	0.0040 (4)	0.0071 (3)	−0.0014 (4)
O1	0.0206 (11)	0.0223 (15)	0.0251 (11)	−0.0019 (10)	−0.0008 (9)	−0.0033 (10)
O2	0.0275 (12)	0.0122 (13)	0.0316 (13)	0.0011 (10)	0.0048 (10)	0.0004 (10)
O3	0.0217 (11)	0.0167 (12)	0.0227 (12)	0.0003 (9)	0.0010 (9)	−0.0035 (10)
N1	0.0276 (14)	0.0291 (18)	0.0228 (14)	−0.0027 (13)	0.0111 (12)	−0.0029 (13)
N2	0.0272 (15)	0.0315 (18)	0.0207 (14)	−0.0017 (13)	0.0071 (12)	0.0004 (13)
N3	0.0167 (13)	0.0118 (14)	0.0244 (14)	−0.0006 (11)	0.0028 (11)	0.0003 (12)
C1	0.0180 (14)	0.0200 (19)	0.0212 (15)	0.0024 (13)	0.0037 (12)	−0.0025 (13)
C2	0.0270 (17)	0.0200 (19)	0.0257 (17)	−0.0012 (14)	0.0093 (14)	−0.0035 (15)
C3	0.0209 (16)	0.023 (2)	0.0247 (17)	−0.0023 (14)	0.0099 (14)	−0.0002 (15)
C4	0.0286 (17)	0.029 (2)	0.0355 (19)	0.0004 (16)	0.0169 (15)	−0.0036 (17)
C5	0.0227 (17)	0.033 (2)	0.039 (2)	0.0037 (16)	0.0057 (15)	0.0045 (18)
C6	0.0254 (17)	0.034 (2)	0.0289 (18)	−0.0038 (16)	0.0022 (15)	0.0032 (17)
C7	0.0180 (15)	0.0166 (18)	0.0217 (16)	0.0020 (13)	0.0037 (13)	0.0001 (14)
C8	0.0185 (15)	0.0191 (18)	0.0176 (15)	0.0009 (13)	0.0066 (13)	0.0010 (13)
C9	0.0248 (15)	0.0193 (19)	0.0221 (15)	−0.0017 (14)	0.0063 (12)	−0.0032 (15)
C10	0.0291 (18)	0.025 (2)	0.0348 (19)	−0.0047 (15)	0.0175 (15)	−0.0048 (16)
C11	0.0272 (18)	0.023 (2)	0.0296 (19)	−0.0005 (15)	0.0042 (15)	−0.0018 (16)
C12	0.0252 (16)	0.027 (2)	0.0249 (17)	−0.0017 (15)	0.0098 (14)	−0.0024 (15)
C13	0.0212 (16)	0.0188 (19)	0.0308 (18)	−0.0029 (14)	0.0091 (14)	−0.0026 (14)
C14	0.0219 (16)	0.0178 (17)	0.0307 (18)	−0.0031 (14)	0.0132 (14)	−0.0008 (15)
C15	0.0252 (17)	0.026 (2)	0.0315 (18)	−0.0002 (15)	0.0122 (15)	0.0014 (16)
C16	0.0263 (18)	0.032 (2)	0.0299 (19)	−0.0056 (16)	0.0104 (15)	−0.0006 (16)
C17	0.0345 (19)	0.032 (2)	0.038 (2)	−0.0106 (17)	0.0241 (17)	−0.0103 (17)
C18	0.0280 (18)	0.0231 (19)	0.052 (2)	0.0039 (16)	0.0233 (17)	0.0039 (18)
C19	0.0279 (17)	0.026 (2)	0.0324 (18)	−0.0028 (16)	0.0156 (14)	0.0031 (16)
O1W	0.0281 (13)	0.0268 (16)	0.0327 (13)	−0.0001 (11)	0.0033 (10)	−0.0008 (11)

### Geometric parameters (Å, °)

**Table:**

S1—C3	1.759 (3)	C9—C11	1.521 (5)
S1—C2	1.809 (3)	C9—C12	1.526 (5)
O1—C1	1.428 (3)	C10—H10A	0.9800
O1—H1O	0.833 (10)	C10—H10B	0.9800
O2—C8	1.219 (4)	C10—H10C	0.9800
O3—C8	1.357 (4)	C11—H11A	0.9800
O3—C9	1.473 (4)	C11—H11B	0.9800
N1—C4	1.343 (4)	C11—H11C	0.9800
N1—C3	1.345 (4)	C12—H12A	0.9800
N2—C3	1.321 (4)	C12—H12B	0.9800
N2—C6	1.349 (5)	C12—H12C	0.9800
N3—C8	1.350 (4)	C13—C14	1.513 (5)
N3—C7	1.458 (4)	C13—H13A	0.9900
N3—H3N	0.856 (10)	C13—H13B	0.9900
C1—C2	1.521 (4)	C14—C15	1.389 (5)
C1—C7	1.533 (4)	C14—C19	1.390 (5)
C1—H1	1.0000	C15—C16	1.383 (5)
C2—H2A	0.9900	C15—H15	0.9500
C2—H2B	0.9900	C16—C17	1.373 (5)
C4—C5	1.373 (5)	C16—H16	0.9500
C4—H4	0.9500	C17—C18	1.381 (5)
C5—C6	1.371 (5)	C17—H17	0.9500
C5—H5	0.9500	C18—C19	1.387 (5)
C6—H6	0.9500	C18—H18	0.9500
C7—C13	1.534 (4)	C19—H19	0.9500
C7—H7	1.0000	O1W—H1W	0.846 (10)
C9—C10	1.520 (5)	O1W—H2W	0.842 (10)
			
C3—S1—C2	102.08 (16)	C10—C9—C12	113.2 (3)
C1—O1—H1O	107 (3)	C11—C9—C12	109.8 (3)
C8—O3—C9	120.2 (2)	C9—C10—H10A	109.5
C4—N1—C3	115.3 (3)	C9—C10—H10B	109.5
C3—N2—C6	114.9 (3)	H10A—C10—H10B	109.5
C8—N3—C7	122.1 (3)	C9—C10—H10C	109.5
C8—N3—H3N	117 (2)	H10A—C10—H10C	109.5
C7—N3—H3N	118 (2)	H10B—C10—H10C	109.5
O1—C1—C2	108.3 (2)	C9—C11—H11A	109.5
O1—C1—C7	107.9 (2)	C9—C11—H11B	109.5
C2—C1—C7	111.3 (3)	H11A—C11—H11B	109.5
O1—C1—H1	109.8	C9—C11—H11C	109.5
C2—C1—H1	109.8	H11A—C11—H11C	109.5
C7—C1—H1	109.8	H11B—C11—H11C	109.5
C1—C2—S1	112.5 (2)	C9—C12—H12A	109.5
C1—C2—H2A	109.1	C9—C12—H12B	109.5
S1—C2—H2A	109.1	H12A—C12—H12B	109.5
C1—C2—H2B	109.1	C9—C12—H12C	109.5
S1—C2—H2B	109.1	H12A—C12—H12C	109.5
H2A—C2—H2B	107.8	H12B—C12—H12C	109.5
N2—C3—N1	127.6 (3)	C14—C13—C7	112.4 (3)
N2—C3—S1	120.6 (3)	C14—C13—H13A	109.1
N1—C3—S1	111.8 (2)	C7—C13—H13A	109.1
N1—C4—C5	122.4 (3)	C14—C13—H13B	109.1
N1—C4—H4	118.8	C7—C13—H13B	109.1
C5—C4—H4	118.8	H13A—C13—H13B	107.9
C6—C5—C4	116.9 (3)	C15—C14—C19	118.5 (3)
C6—C5—H5	121.5	C15—C14—C13	121.7 (3)
C4—C5—H5	121.5	C19—C14—C13	119.7 (3)
N2—C6—C5	123.0 (3)	C16—C15—C14	120.6 (3)
N2—C6—H6	118.5	C16—C15—H15	119.7
C5—C6—H6	118.5	C14—C15—H15	119.7
N3—C7—C1	109.9 (2)	C17—C16—C15	120.4 (3)
N3—C7—C13	109.6 (2)	C17—C16—H16	119.8
C1—C7—C13	111.4 (3)	C15—C16—H16	119.8
N3—C7—H7	108.6	C16—C17—C18	119.9 (3)
C1—C7—H7	108.6	C16—C17—H17	120.0
C13—C7—H7	108.6	C18—C17—H17	120.0
O2—C8—N3	125.5 (3)	C17—C18—C19	119.8 (3)
O2—C8—O3	125.3 (3)	C17—C18—H18	120.1
N3—C8—O3	109.3 (3)	C19—C18—H18	120.1
O3—C9—C10	109.6 (2)	C18—C19—C14	120.8 (3)
O3—C9—C11	102.2 (3)	C18—C19—H19	119.6
C10—C9—C11	110.7 (3)	C14—C19—H19	119.6
O3—C9—C12	110.9 (2)	H1W—O1W—H2W	107 (4)
			
O1—C1—C2—S1	65.2 (3)	C7—N3—C8—O2	15.8 (5)
C7—C1—C2—S1	−176.4 (2)	C7—N3—C8—O3	−165.2 (3)
C3—S1—C2—C1	89.9 (2)	C9—O3—C8—O2	−3.5 (5)
C6—N2—C3—N1	1.4 (5)	C9—O3—C8—N3	177.5 (2)
C6—N2—C3—S1	−179.2 (3)	C8—O3—C9—C10	70.1 (3)
C4—N1—C3—N2	−0.6 (5)	C8—O3—C9—C11	−172.5 (3)
C4—N1—C3—S1	179.9 (2)	C8—O3—C9—C12	−55.5 (4)
C2—S1—C3—N2	−0.9 (3)	N3—C7—C13—C14	−70.4 (3)
C2—S1—C3—N1	178.6 (2)	C1—C7—C13—C14	167.8 (3)
C3—N1—C4—C5	0.1 (5)	C7—C13—C14—C15	91.7 (4)
N1—C4—C5—C6	−0.5 (5)	C7—C13—C14—C19	−86.0 (4)
C3—N2—C6—C5	−1.7 (5)	C19—C14—C15—C16	1.2 (5)
C4—C5—C6—N2	1.4 (6)	C13—C14—C15—C16	−176.5 (3)
C8—N3—C7—C1	−135.6 (3)	C14—C15—C16—C17	−0.7 (5)
C8—N3—C7—C13	101.7 (3)	C15—C16—C17—C18	−0.5 (5)
O1—C1—C7—N3	−179.3 (3)	C16—C17—C18—C19	1.1 (5)
C2—C1—C7—N3	62.0 (3)	C17—C18—C19—C14	−0.6 (5)
O1—C1—C7—C13	−57.7 (3)	C15—C14—C19—C18	−0.5 (5)
C2—C1—C7—C13	−176.3 (3)	C13—C14—C19—C18	177.3 (3)

### Hydrogen-bond geometry (Å, °)

**Table:**

*D*—H···*A*	*D*—H	H···*A*	*D*···*A*	*D*—H···*A*
O1—H1o···O1w^i^	0.83 (2)	2.00 (2)	2.813 (4)	168 (4)
O1w—H1w···N1^ii^	0.85 (2)	2.12 (2)	2.958 (4)	174 (4)
O1w—H2w···O1	0.84 (3)	2.06 (3)	2.893 (4)	170 (4)
N3—H3n···O2^i^	0.859 (19)	2.126 (16)	2.910 (3)	152 (3)

Symmetry codes: (i) *x*, *y*+1, *z*; (ii) −*x*+1, *y*−1, −*z*+2.
